# FOLFOX plus nivolumab combination therapy for cisplatin-unfit patients with untreated advanced esophageal squamous cell carcinoma

**DOI:** 10.1007/s10388-026-01227-x

**Published:** 2026-06-10

**Authors:** Nozomu Ogura, Shun Yamamoto, Ryuichi Morita, Hiroshi Imazeki, Hideaki Takahashi, Yoshitaka Honma, Tairo Kashihara, Daisuke Kurita, Koshiro Ishiyama, Hiroyuki Daiko, Yasuyuki Seto, Ken Kato

**Affiliations:** 1 Department of Head and Neck, Esophageal Medical Oncology, National Cancer Center Hospital, 5-1-1 Tsukiji, Chuo-ku, Tokyo, 1040045 Japan; 2 Department of Radiation Oncology, National Cancer Center Hospital, Tokyo, Japan; 3 Department of Esophageal Surgery, National Cancer Center Hospital, Tokyo, Japan

**Keywords:** Advanced esophageal squamous cell carcinoma, Cisplatin-unfit, FOLFOX, Nivolumab

## Abstract

**Background:**

Combination chemotherapy with cisplatin and fluorouracil plus an immune checkpoint inhibitor (ICI) is a standard first-line treatment for advanced esophageal squamous cell carcinoma (ESCC). However, cisplatin is unsuitable for patients with renal or cardiac dysfunction and older patients because of nephrotoxicity and the need for hydration. Oxaliplatin-based regimens (e.g., FOLFOX) have demonstrated clinically meaningful efficacy and are well tolerated in cisplatin-unfit patients with untreated advanced ESCC. This study investigated the effectiveness and safety of FOLFOX combined with an ICI in these patients.

**Methods:**

Cisplatin-unfit patients with untreated advanced ESCC who received FOLFOX plus nivolumab at our institution between January 2024 and July 2025 were retrospectively identified. Progression-free survival, overall survival, and the response rate were evaluated as efficacy endpoints and adverse events as safety endpoints.

**Results:**

Seventeen patients with a median age of 75 years (range, 62–82) were included. ECOG performance status was 0 in 3 cases (17.6%), 1 in 11 (64.7%), and 2 in 3 (17.6%). The main reasons for cisplatin ineligibility were older age (88.2%) and poor condition (58.8%). The response rate was 57.1% in patients with measurable lesions during a median follow-up of 9.9 months. Median progression-free survival was 6.5 months (95% confidence interval 3.6–not reached [NR]) and median overall survival was 15.6 months (4.5–NR). Common adverse events were neutropenia (41.2%) and leukopenia (35.3%). No treatment-related deaths occurred.

**Conclusions:**

FOLFOX plus nivolumab might have effectiveness and safety comparable with that of cisplatin and fluorouracil plus nivolumab in cisplatin-unfit patients with untreated advanced ESCC.

**Supplementary Information:**

The online version contains supplementary material available at https://doi.org/10.1007/s10388-026-01227-x.

## Introduction

Esophageal cancer is the eighth leading type of cancer and the seventh most common cause of cancer-related deaths worldwide [1]. Moreover, it is the ninth most common cancer in men and accounts for 3% of cancer-related deaths in Japan [2]. Esophageal cancer has mainly two histological subtypes, namely, esophageal squamous cell carcinoma (ESCC) and esophageal adenocarcinoma. ESCC is the main histological subtype worldwide and accounts for about 90% of the burden of esophageal cancer in Japan. The major risk factors for ESCC are alcohol consumption and smoking [3].

Palliative chemotherapy is the standard treatment for patients with unresectable advanced ESCC [4]. According to the results of the CheckMate 648 [5], KEYNOTE-590 [6] and RATIONALE-306 trials [7], combination therapy containing cisplatin and fluorouracil (CF) plus an immune checkpoint inhibitor (ICI) is a standard first-line treatment for patients with untreated advanced ESCC. However, these pivotal trials exclusively enrolled patients considered fit for cisplatin-based chemotherapy, which limits the applicability of their results to real-world clinical practice. In routine clinical practice, a substantial proportion of patients are deemed unfit for cisplatin because of advanced age, malnutrition, impaired renal or cardiac function, or poor performance status.

Oxaliplatin is a third-generation platinum agent with less renal toxicity than cisplatin and does not require hydration. The FOLFOX regimen has shown clinical meaningful efficacy and safety in cisplatin-unfit patients with advanced ESCC [8, 9] and is now used in clinical practice. Therefore, an oxaliplatin-based regimen such as FOLFOX plus an ICI might be an option for these patients. However, data regarding the real-world effectiveness and safety of FOLFOX combined with an ICI as first-line treatment for cisplatin-unfit patients with advanced ESCC remain limited.

To address this gap in the literature, we evaluated the real-world effectiveness and safety of combination therapy containing FOLFOX plus nivolumab (FOLFOX + Nivo) as a first-line treatment for cisplatin-unfit patients with untreated advanced ESCC.

## Methods

### Patients

This was a retrospective, single-center, observational study. The study included patients who were identified to have had histologically confirmed ESCC, clinical stage IVB according to the UICC-TNM 8th edition or recurrent disease, and treatment with FOLFOX + Nivo as first-line palliative treatment at our institution between January 2024 and July 2025. First-line treatment was defined as no previous palliative chemotherapy for advanced ESCC or recurrent disease, administered more than 6 months after the last dose of perioperative treatment. Cisplatin-unfit was assessed using the following criteria: renal dysfunction indicated by a creatinine clearance of < 60 mL/min; cardiac dysfunction indicated by comorbid heart disease and/or abnormal findings on echocardiography; age ≥ 70 years: poor condition due to other comorbidities. These were used as reference criteria, and the final determination was made by the physician based on the overall clinical context. The study was approved by the Institutional Review Board of the National Cancer Center Hospital (approval number 2020 − 287) and conducted in accordance with the principles of the Declaration of Helsinki. The requirement for informed consent was waived because of the retrospective design of the study.

### Treatment

FOLFOX + Nivo consisted of oxaliplatin 85 mg/m², a bolus of fluorouracil 400 mg/m², leucovorin 200 mg/m², and nivolumab 240 mg on day 1, and a continuous infusion of fluorouracil 2400 mg/m² for 46 h, repeated every 2 weeks until disease progression, intolerable toxicity, or patient refusal to continue with treatment. Oxaliplatin in the FOLFOX regimen was often planned to be discontinued after 6 cycles to mitigate cumulative chemotherapy-induced peripheral neuropathy. To assess the intensity of FOLFOX + Nivo treatment, relative dose intensity was calculated as the ratio of the actual dose intensity to the planned dose intensity. The early discontinuation was defined as discontinuation within 6 cycles of FOLFOX + Nivo.

### Evaluations and statistical analysis

For evaluation of efficacy, OS was defined as the interval between the date of initiation of treatment and the date of death from any cause or the date of censoring at the last time the subject was known to be alive, and progression-free survival (PFS) was defined as the interval between the date of initiation of treatment and the date of progression, recurrence, or death from any cause or the date of censoring at the last time the subject was known to be alive. The date of progression was defined as the date of radiological progression or the date of clinical disease progression when imaging findings were inconclusive and progression was determined by the treating physician. The duration of response (DOR) was defined as the interval between the first documented objective response (complete or partial) and the date of disease progression or death from any cause. The response rate was calculated according to the Response Evaluation Criteria in Solid Tumors (version 1.1). The best response was analyzed while patients were receiving FOLFOX + Nivo for target lesions. The timing of the evaluation was not specified. PFS, overall survival (OS), and DOR were estimated by the Kaplan–Meier method, and the confidence interval (CI) was calculated according to the Greenwood formula. The CI for median survival time was calculated using the Brookmeyer–Crowley method. Hazard ratios (HRs) were obtained using Cox regression models. Adverse events were evaluated according to the Common Terminology Criteria for Adverse Events (CTCAE) version 5.0, as translated by the Japan Clinical Oncology Group. All data were analyzed using EZR version 1.68 (R Foundation for Statistical Computing, Vienna, Austria).

## Results

### Patient characteristics

Seventeen patients met the study selection criteria. They included 16 men and 1 woman. The median age was 75 years (range, 62–82). Performance status (PS) was 0 in 3 patients (17.6%), 1 in 11 (64.7%), and ≥ 2 in 3 (17.6%) at the time of initiation of FOLFOX + Nivo. Ten patients (58.8%) had cStage IVB disease and 7 (41.2%) had recurrent disease. The most common reasons for being unfit for cisplatin were older age (88.2%) and poor condition (58.8%). The programmed cell death-ligand 1 (PD-L1) combined positive score (CPS) was ≥ 10 in 7 patients (41.2%) and < 10 in 8 (47.1%). The PD-L1 tumor proportion score (TPS) was ≥ 1% in 7 patients (41.2%) and < 1% in 8 (47.1%) (Table 1). The relative dose intensity of FOLFOX + Nivo is shown in supplementary Table 1. Early discontinuation rate was 41.2% (*n* = 7).

**Table 1:** Patient demographics and clinical characteristics

	*n* = 17	*n* = 14*
Age (years), median (range)	75 (62–82)	75 (62–82)
Sex (%)		
Male/female	16 (94.1)/1 (5.9)	13 (92.9)/1 (7.1)
ECOG PS (%)		
0/1/≥2	3 (17.6)/11 (64.7)/3 (17.6)	2 (14.3)/9 (64.3)/3 (21.4)
Smoking history		
Yes/No	15 (88.2)/2 (11.8)	12 (85.7)/2 (14.3)
Prior radiation therapy		
Yes/No	2 (11.8)/15 (88.2)	1 (7.1)/13 (92.9)
Reason for cisplatin-unfit (%)**		
Renal dysfunction/cardiac dysfunction/elderly/poor condition	9 (52.9)/1 (5.9)/15 (88.2)/10 (58.8)	7 (50.0)/1 (7.1)/13 (92.9)/9 (64.3)
Disease status (%)		
Metastatic/recurrent	10 (58.8)/7 (41.2)	9 (64.3)/5 (35.7)
Location of primary tumor (%)		
Ce/Ut/Mt/Lt	5 (29.4)/3 (17.6)/5 (29.4)/4 (23.5)	4 (28.6)/3 (21.4)/4 (28.6)/3 (21.4)
PD-L1 tumor proportion score (%)		
≥1%/<1%/not evaluated	10 (58.8)/5 (29.4)/2 (11.8)	8 (57.1)/4 (28.6)/2 (14.3)
PD-L1 combined positive score (%)		
≥10/1–9/<1/not evaluated	7 (41.2)/5 (29.4)/3 (17.6)/2 (11.8)	6 (42.9)/4 (28.6)/2 (14.3)/2 (14.3)
Site of metastasis (%)**		
Lymph node/lung/liver/bone	16 (94.1)/5 (29.4)/3 (17.6)/1 (5.9)	13 (92.9)/ 5 (35.7)/3 (21.4)/1 (7.1)
Metastatic sites, n (%)		
1/≥2	9 (52.9)/8 (47.1)	6 (42.9)/8 (57.1)
Previous treatment (%)		
Surgery/radiotherapy/chemotherapy***/none	7 (41.2)/0 (0.0)/6 (35.3)/10 (58.8)	5 (35.7)/0 (0.0)/5 (35.7)/9 (64.3)

*Ce* cervical esophagus, *ECOG PS* Eastern Cooperative Oncology Group performance status, *Lt* lower thoracic esophagus, *Mt* middle thoracic esophagus, *PD-L1* programmed cell death-ligand 1, *Ut* upper thoracic esophagus

*Patients with measurable lesions

**Duplicated

***Perioperative treatments

### Effectiveness

Among 14 patients with measurable lesions, 8 (57.1%) showed a partial response and 3 (21.4%) showed stable disease. Therefore, the response rate was 57.1% and the disease control rate was 78.6%. In terms of PD-L1 expression, the respective response and disease control rates were 66.7% and 83.3% in the population with a PD-L1 CPS of ≥ 10, 50.0% and 75.0% in that with a PD-L1 CPS of < 10 and with a CPS < 1 or not evaluable (NE), 50.0% and 62.5% in that with a PD-L1 TPS of ≥ 1%, and 66.7% and 100.0% in that with a PD-L1 TPS of < 1% or NE (Table 2).

**Table 2:** Best response during treatment

	PD-L1 TPS		PD-L1 CPS			Overall *n* = 14 (%)*
	≥ 1% *n* = 8 (%)	< 1% or NE *n* = 6 (%)	≥ 10 *n* = 6 (%)	1–9 *n* = 4 (%)	< 1 or NE *n* = 4 (%)	
CR	0 (0.0)	0 (0.0)	0 (0.0)	0 (0.0)	0 (0.0)	0 (0.0)
PR	4 (50.0)	4 (66.7)	4 (66.7)	2 (50.0)	2 (50.0)	8 (57.1)
SD	1 (12.5)	2 (33.3)	1 (16.7)	1 (25.0)	1 (25.0)	3 (21.4)
PD	3 (37.5)	0 (0.0)	1 (16.7)	1 (25.0)	1 (25.0)	3 (21.4)
NE	0 (0.0)	0 (0.0)	0 (0.0)	0 (0.0)	0 (0.0)	0 (0.0)
Response rate (%)	50.0	66.7	66.7	50.0	50.0	57.1
Disease control rate (%)	62.5	100.0	83.3	75.0	75.0	78.6

*CPS* combined positive score, *CR* complete response, *NE* not evaluable, *PD* progressive disease, *PD-L1* programmed cell death-ligand 1, *PR* partial response, *SD* stable disease, *TPS* tumor proportion score

*Three subjects were excluded from the best overall response analysis because they had no measurable lesions

**Fig. 1:**
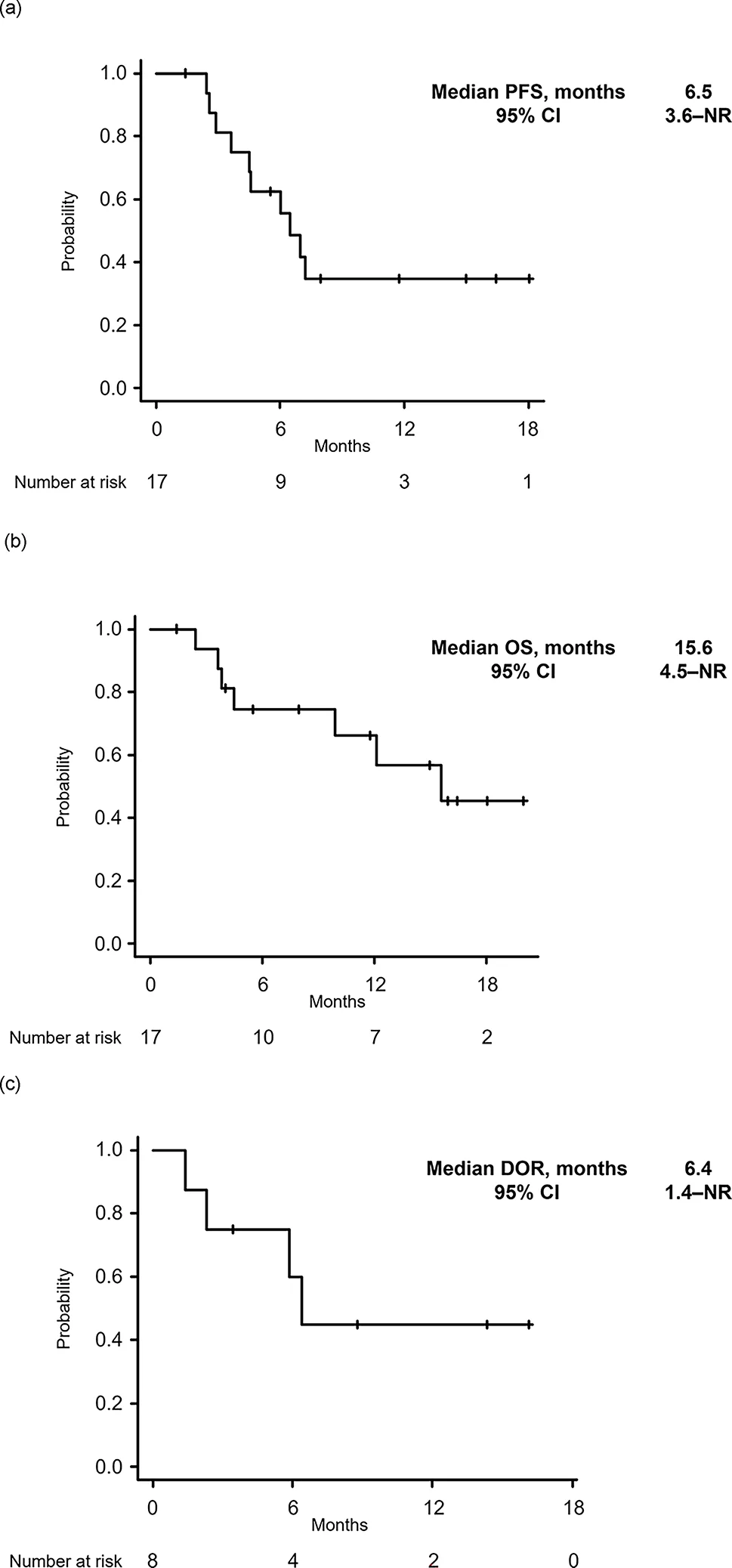
Median progression-free survival, overall survival, and duration of response in the overall study population. **a** Progress-free survival. **b** Overall survival. **c** Duration of response. *CI* confidence interval, *DOR* duration of response, *NR* not reached, *OS* overall survival, *PFS* progression-free survival

During a median follow-up of 9.9 months, the median PFS was 6.5 months (95% CI 3.6–not reached [NR]), and the 12-month PFS rate was 34.7% (95% CI 13.0–57.8) (Fig. 1a). The median OS was 15.6 months (95% CI 4.5–NR), and the 12-month OS rate was 66.2% (95% CI 36.3–84.6) (Fig. 1b). Among patients who achieved an objective response, the median DOR was 6.4 months (95% CI 1.4–NR) (Fig. 1c).

**Fig. 2:**
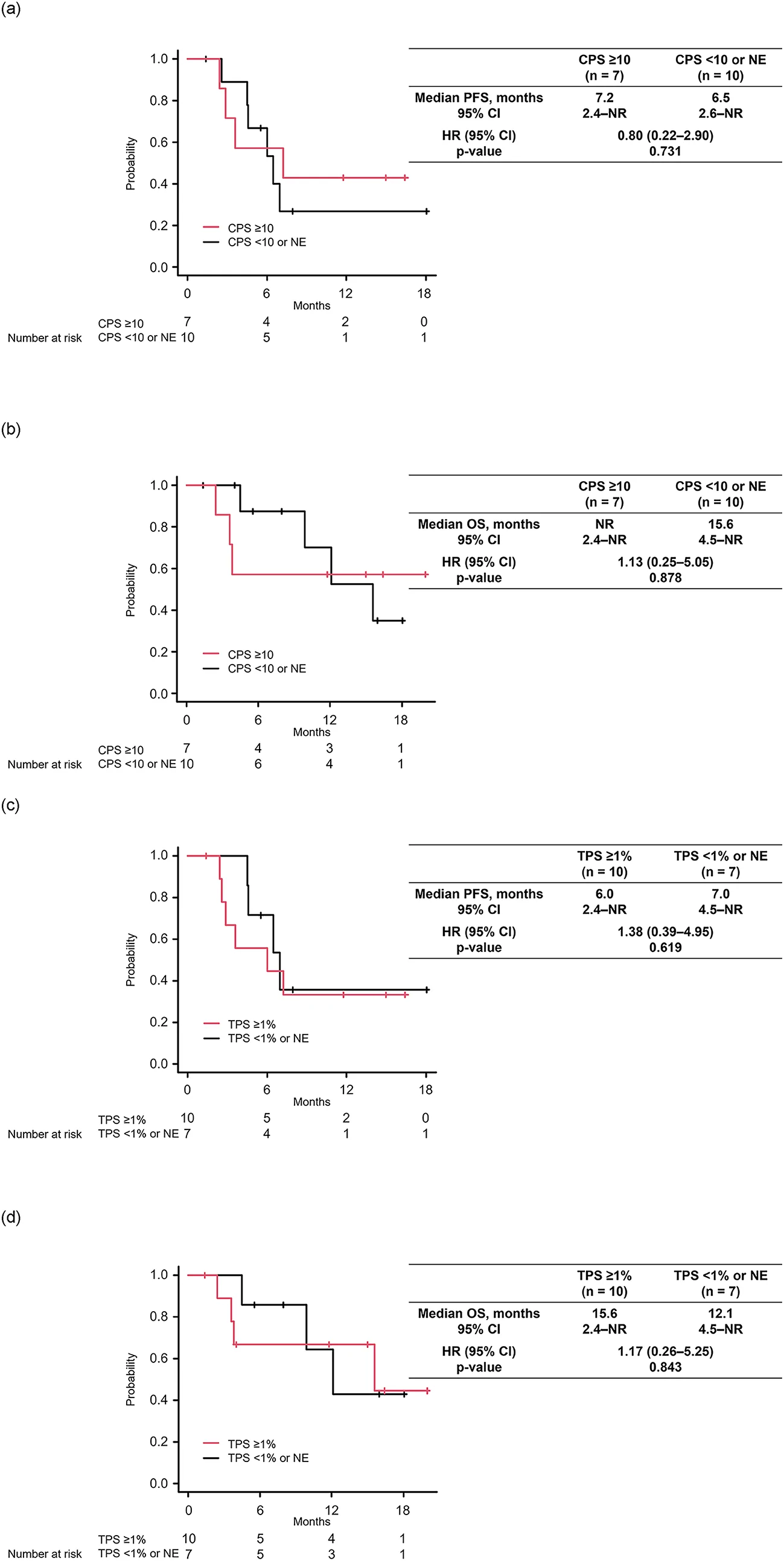
Median progression-free survival and overall survival according to PD-L1 CPS and TPS status. **a** Progression-free survival by PD-L1 CPS. **b** Overall survival by PD-L1 CPS. **c** Progression-free survival by PD-L1 TPS. **d** Overall survival by PD-L1 TPS. *CI* confidence interval, *CPS* combined positive score, *HR* hazard ratio, *NE* not evaluated, *NR* not reached, *OS* overall survival, *PD-L1* programmed cell death-ligand 1, *PFS* progression-free survival, *TPS* tumor proportion score

Median PFS was 7.2 months in the population with a PD-L1 CPS of ≥ 10 and 6.5 months in that with a PD-L1 CPS of < 10; the difference was not statistically significant (HR 0.80, 95% CI 0.22–2.90) (Fig. 2a). Median OS was not reached in the population with a PD-L1 CPS of ≥ 10 and was 15.6 months in that with a PD-L1 CPS of < 10; again, this finding did not reach statistical significance (HR 1.13, 95% CI 0.25–5.05) (Fig. 2b). Median PFS was 6.0 months when the TPS was ≥ 1% and 7.0 months when it was < 1%; the difference was not statistically significant (HR 1.38, 95% 0.39–4.95) (Fig. 2c). Median OS was 15.6 months when the TPS was ≥ 1% and 12.1 months when it was < 1%, and the difference did not reach statistical significance (HR 1.17, 95% CI 0.26–5.25) (Fig. 2d).

### Safety

The incidence of all-grade adverse events was 70.6% and that of grade ≥ 3 adverse events was 41.2%. The most common all-grade adverse events were neutropenia (41.2%), followed by leukopenia (35.3%), anemia (23.5%), and pneumonitis (23.5%). The most common grade ≥ 3 adverse events were neutropenia (29.4%), leukopenia (11.8%), and pneumonitis (11.8%).

Immune-related adverse events (irAEs) were observed in 47.1% of patients, the most being pneumonitis (17.6%) and rash (17.6%) followed by adrenal insufficiency (11.8%). Grade ≥ 3 irAEs included pneumonitis (11.8%) and rash (5.9%). No grade 5 irAEs were observed during first-line treatment (Table 3).

**Table 3:** Adverse events during first-line treatment*

	Patients, *n* (%)					
	Grade 1	Grade 2	Grade 3	Grade 4	Grade ≥ 3	Any grade
Hematologic toxicity						
Leukopenia	1 (5.9)	3 (17.6)	2 (11.8)	0 (0.0)	2 (11.8)	6 (35.3)
Neutropenia	0 (0.0)	2 (11.8)	3 (17.6)	2 (11.8)	5 (29.4)	7 (41.2)
Anemia	3 (17.6)	1 (5.9)	0 (0.0)	0 (0.0)	0 (0.0)	4 (23.5)
Thrombocytopenia	2 (17.6)	1 (5.9)	0 (0.0)	0 (0.0)	0 (0.0)	3 (17.6)
Non-hematologic toxicity						
Nausea	3 (17.6)	0 (0.0)	0 (0.0)	0 (0.0)	0 (0.0)	3 (17.6)
Peripheral neuropathy	1 (5.9)	1 (5.9)	0 (0.0)	0 (0.0)	0 (0.0)	2 (11.8)
Febrile neutropenia	-	-	0 (0.0)	0 (0.0)	0 (0.0)	0 (0.0)
Pneumonitis	1 (5.9)	1 (5.9)	2 (11.8)	0 (0.0)	2 (11.8)	4 (23.5)
Rash	1 (5.9)	1 (5.9)	1 (5.9)	0 (0.0)	1 (5.9)	3 (17.6)
Adrenal insufficiency	0 (0.0)	2 (11.8)	0 (0.0)	0 (0.0)	0 (0.0)	2 (11.8)
Hypothyroidism	0 (0.0)	1 (5.9)	0 (0.0)	0 (0.0)	0 (0.0)	1 (5.9)
Uveitis	0 (0.0)	1 (5.9)	0 (0.0)	0 (0.0)	0 (0.0)	1 (5.9)
Stomatitis	0 (0.0)	0 (0.0)	0 (0.0)	0 (0.0)	0 (0.0)	0 (0.0)
Diarrhea	0 (0.0)	0 (0.0)	0 (0.0)	0 (0.0)	0 (0.0)	0 (0.0)

*There were no grade 5 adverse events

## Discussion

This retrospective study evaluated the real-world effectiveness and safety of FOLFOX + Nivo as first-line treatment for cisplatin-unfit patients with advanced ESCC. In our cohort, FOLFOX + Nivo was associated with encouraging survival outcomes and an acceptable safety profile. Furthermore, the short-term and long-term outcomes of FOLFOX + Nivo were promising regardless of PD-L1 CPS and TPS status.

In the CheckMate 648, KEYNOTE-590, and RATIONALE-306 trials, median PFS was 5.8, 6.3, and 7.3 months, respectively, and median OS was 13.2, 12.6, and 17.2 months in patients with advanced ESCC who received CF combined with an ICI [5–7]. Although direct comparisons across trials are inappropriate, the median PFS and OS of 6.5 months and 15.6 months observed in the present study appears numerically within the range reported in these trials, despite the inclusion of an exclusively cisplatin-unfit population. This observation suggests that FOLFOX + Nivo may provide clinically meaningful disease control in selected patients who are unfit for cisplatin. The effectiveness of FOLFOX + Nivo also tended to be better in our study than in previous reports on FOLFOX as first-line treatment for patients with advanced ESCC who were cisplatin-unfit. A prospective observational study in Japan reported a response rate of 22.6%, a median PFS of 3.9 months, and a median OS of 13.3 months in cisplatin-unfit patients with advanced ESCC during a median follow-up of 20.2 months [8]. Another retrospective multicenter study in Japan reported a response rate of 35%, a median PFS of 3.8 months, and a median OS of 13.9 months during a median follow-up of 14.7 months [9]. Oxaliplatin is known to induce immunogenic cell death in gastric cancer [10]. This biological property provides a plausible rationale for combining oxaliplatin-based chemotherapy with ICIs. Although cross-trial comparisons must be interpreted with caution, these data indicate that FOLFOX + Nivo may provide at least an additive effect. The recent LEAP-014 trial included patients with untreated advanced ESCC who received a combination of FOLFOX or CF plus pembrolizumab as a control arm (doublet chemotherapy plus pembrolizumab) and reported a median OS of 15.5 months, a median PFS of 6.9 months, and an objective response rate of 54.8% [11]. In the population that received a FOLFOX-based regimen, the HR for OS was reported to be 0.85 (95% CI 0.67–1.06), which is slightly more favorable than that found with other regimens. Furthermore, the LEAP-014 included mainly cisplatin-fit patients, which made it difficult to compare its efficacy results directly with those of FOLFOX + Nivo in our retrospective analysis. However, FOLFOX + Nivo has effectiveness in cisplatin-unfit patients potentially similar to that of doublet chemotherapy plus ICI in their cisplatin-fit counterparts. In exploratory subgroup analyses, no clear association between PD-L1 CPS or TPS and clinical outcomes was observed. However, larger prospective studies are required to clarify the role of PD-L1 status in this setting.

Our data for FOLFOX + Nivo do not provide any new safety signals in patients with advanced ESCC. In our study, neutropenia was the most common adverse hematological event. In previous reports on FOLFOX as the first-line treatment, any grade/grade ≥ 3 neutropenia was reported to be 60.0%/26.7% in the prospective study [8] and 35%/23% in the retrospective study [9]. The characteristic non-hematological adverse events related to FOLFOX were stomatitis and neuropathy; any grade/grade ≥ 3 adverse events were reported at 13.3%/0% for stomatitis and 46.7%/3.3% for neuropathy in the prospective study [8] and NR/NR and 48%/0%, respectively, in the retrospective study. Furthermore, the CheckMate 648 trial of CF plus nivolumab reported a treatment-related adverse event rate of 96% for any grade and 47% for grade ≥ 3, with frequent all-grade irAEs (gastrointestinal, 21%; rash, 17%; endocrine, 12%) [5]. The incidence of pneumonitis appeared slightly higher than that reported in some previous trials, which may reflect the advanced age and comorbidity burden of the study population, as well as pulmonary toxicity of nivolumab and oxaliplatin. In fact, previous studies have identified advanced age and underlying pulmonary conditions as risk factors for irAE pneumonitis [12]. In our study, oxaliplatin in the FOLFOX was often planned to be discontinued after 6 cycles and oxaliplatin exposure was limited in many cases, suggesting that oxaliplatin was less likely to be the primary driver of pneumonitis. In addition, the study population usually had a smoking history (88.2%) or prior radiation therapy (5.9%), these factors might affect the incidence of pneumonitis. As these adverse events were previously reported and the overall incidence of adverse events in the present study tended to be lower than in previous reports, the safety profile of FOLFOX + Nivo might be manageable in clinical practice.

This study has several limitations, in particular a single-center retrospective design with a small number of patients and a short observation period of about 10 months. Cisplatin-unfit was determined ultimately based on the physician’s judgment, which might limit reproducibility and cause selection bias. Phase II studies are ongoing in Japan to evaluate FOLFOX + Nivo as first-line treatment for patients with untreated advanced ESCC (jRCTs 031230630 and 051240108-2). Their results might strengthen the value of FOLFOX + Nivo. Furthermore, there were no comparative data between FOLFOX + Nivo and combination therapy containing nivolumab plus ipilimumab as first-line treatment for cisplatin-unfit patients with untreated advanced ESCC. In clinical practice in Japan, some cisplatin-unfit patients, including those who would otherwise be candidates for FOLFOX + Nivo, may receive combination therapy with nivolumab plus ipilimumab. Therefore, there is a need for direct comparisons between FOLFOX + Nivo and nivolumab plus ipilimumab.

In conclusion, this retrospective single-center analysis suggests that combination therapy with FOLFOX + Nivo may be a feasible treatment option for patients with untreated advanced ESCC who were unfit for cisplatin, showing encouraging signals of effectiveness and acceptable tolerability.

## Supplementary Information

Below is the link to the electronic supplementary material.

Supplementary Material 1

## Data Availability

The data that support the findings of this study are available from the corresponding author upon reasonable request.
