# Supplementary material for: FOLFOX plus nivolumab combination therapy for cisplatin-unfit patients with untreated advanced esophageal squamous cell carcinoma
Source: Esophagus. 2026 Jun 10;23(4):861–8. doi: 10.1007/s10388-026-01227-x (PMC13723493; doi:10.1007/s10388-026-01227-x)
Supplement: Supplementary file 1 [file 10388_2026_1227_MOESM1_ESM.docx]

Supplementary Table 1. Treatment exposure

|  | 5-FU (Continuous) | L-OHP | Nivolumab |
| --- | --- | --- | --- |
| Median RDI* (range) | 0.86 (0.41–1.02) | 0.79 (0.36–1.02) | 0.93 (0.75–1.02) /  0.91 (0.75–1.02)** |
| Median cycles (range) | 4 (1–12) | 4 (1–7) | 7 (1–19) |
| Reduced starting dose (%) | 6 (35.3) | 7 (41.2) | 0 (0.0) |

*Within the first 6 cycles. **Across all administered cycles.

5-FU, 5-fluorouracil; L-OHP, oxaliplatin; RDI, relative dose intensity.

Supplementary Table 2. Subsequent therapies

|  | FOLFOX + Nivo  n = 17 |
| --- | --- |
| **Continuation** | 4 (23.5) |
| **Observation** | 2 (11.8) |
| **Paclitaxel** | 5 (29.4) |
| **Chemoradiotherapy** | 1 (5.9) |
| **Radiotherapy alone** | 1 (5.9) |
| **Best supportive care** | 4 (23.5) |

Nivo, nivolumab
